# Surgical Outcomes of Minimally Invasive Lung Resection in Nonagenarians: A Retrospective Analysis of Seven Patients at a Single Institution

**DOI:** 10.7759/cureus.82186

**Published:** 2025-04-13

**Authors:** Takahiro Karasaki, Sakashi Fujimori, Souichiro Suzuki, Shinichiro Kikunaga, Yosuke Hamada, Shusei Mihara

**Affiliations:** 1 Department of Thoracic Surgery, Respiratory Center, Toranomon Hospital, Tokyo, JPN

**Keywords:** isolated lung metastasis, minimally invasive surgery, nonagenarians, non small cell lung cancer, video-assisted thoracoscopic surgery (vats)

## Abstract

Introduction

With the global trend of aging populations, the number of nonagenarians diagnosed with malignancies, including lung cancer, is increasing. Despite advancements in minimally invasive surgical techniques, lung resection for nonagenarians remains rare due to concerns regarding comorbidities and surgical risks. This study evaluates the surgical outcomes of lung resection in nonagenarians and introduces a holistic assessment approach to optimize patient care.

Methods

A retrospective review of surgical records from January 2011 to December 2022 identified seven nonagenarians who underwent lung resection under 3-port video-assisted thoracoscopic surgery (VATS). Patient characteristics, surgical details, and postoperative outcomes were analyzed. To holistically evaluate each patient, multifaceted surgical tolerance and prognostic factors were summarized and visualized in a radar plot.

Results

The study cohort consisted of four males and three females aged 90 to 96 years. Wedge resection was performed in six patients, and one patient underwent lobectomy. Mediastinal lymph node dissection was not performed. The median duration of chest tube insertion was two days, and 86% of patients were discharged within one week. Postoperative complications were minimal, with one case of delirium and no occurrences of pneumonia. All patients were discharged in stable condition without deterioration of their activities of daily living. The median overall survival was 4.1 years. One patient succumbed to lung cancer progression, while the remaining patients exhibited favorable long-term survival without recurrence, including one patient whose lung tumor was a metastasis from colorectal cancer. As depicted in the radar plots, all patients had at least one risk factor other than their age.

Conclusion

Lung resection under a minimally invasive approach is feasible for carefully selected nonagenarians, yielding favorable short- and long-term outcomes. Because super-elderlies likely harbor multiple comorbidities, a holistic assessment of each patient is important for personalized patient care.

## Introduction

Populations are aging in many countries around the world. Some countries, including Japan, are becoming super-aging societies with increasing numbers and fractions of super-elderly people. According to the Statistics Bureau of Japan, the percentage of nonagenarians in their 90s in the Japanese population has nearly tripled from 0.8% to 2.3% over the last 20 years [1]. One of the challenges in such a super-aging society is medical care for super elderly individuals, including malignant diseases. The number of elderly patients with malignancy is also increasing. In Japan, the cancer incidence in people aged 85 years or more has increased from 51,699 cases per year in 2000 to 148,083 in 2020, while the incidence rates have not changed from 2,312 cases per 100,000,000 population in 2000 to 2,414 cases in 2020 [2-4]. In the management of patients with cancer, surgery remains the mainstream for providing local control and cure.

High variance across super-elderly patients regarding the activity of daily living (ADL), systemic and cognitive functions, and social environment necessitates a personalized treatment plan for each patient. An advanced age itself does not contraindicate invasive treatment, and favorable surgical outcomes of lung resection in octogenarians have been described elsewhere [5]. However, lung resection for super elderly individuals, especially for nonagenarians, has been rarely performed, possibly due to their comorbidities [6-8]. In the National Cancer Database from 2004 to 2014, 7205 patients with non-small cell lung cancer (NSCLC) aged 90 years or older had complete, clinically relevant data. The majority (n = 4152, 57.6%) of the 7205 nonagenarian patients with stage I-IV NSCLC did not receive any therapy. While the most common therapy used was radiation, only 266 patients (3.7%) were treated with surgery [6]. Nonagenarians undergoing surgery are an under-represented population who are often excluded from aggressive surgical interventions due to perceived risks. On the other hand, the recent advances in minimally invasive surgical techniques have broadened surgical indications for elderly patients. This study aimed to unveil the feasibility and outcomes of lung resection for nonagenarians. We also discuss the conceptual framework of holistic evaluation for super-elderlies to assess and visualize surgical risks.

## Materials and methods

We retrospectively reviewed the surgical records and patient charts of individuals who underwent lung resection in our institution between January 2011 and December 2022. The study included consecutive nonagenarians, i.e., the patients who were 90 years or older, at the time of surgery. Baseline characteristics (past medical history, smoking history, performance status, blood test, pulmonary function test, and other physiological exams), detailed information of surgery (operation time, blood loss, presence of adhesion, and extent of resection), and postoperative disease courses (adverse events after surgery and survival outcomes) were curated using electronic charts and operation notes.

At our institution, we apply the confronting setup for VATS procedures. The patient is positioned in the lateral decubitus position under general anesthesia with double-lumen endotracheal intubation. Two monitors are placed at the cranial end of the patient, with one oriented upside-down. The VATS procedure is carried out using three ports. The primary surgeon stands on the right side of the patient, irrespective of the side being operated on, while the thoracoscope is handled by an assistant surgeon positioned on the patient's left side.

The Charlson’s Comorbidity Index (CCI) was applied to categorize the patients based on their comorbidities [9]. In brief, CCI encompasses 19 medical conditions, and the sum of the weighted index scores is grouped into four risks related to prognosis: low (score 0), medium (1-2), high (3-4), and very high (5 or more). For the assessment of inflammation and nutrition, the Glasgow Prognostic Score (GPS), a scoring system that uses serum C-reactive protein (CRP) and albumin levels, was used [10].

Alternatively, for the assessment of malnutrition, the Geriatric Nutrition Risk Index, a scoring system that uses serum albumin levels, body heights, and weights, was also applied [11]. Respiratory function was assessed using a spirometry test. The severity of obstructive disorder was categorized into five groups: no obstructive disorder (forced expiratory volume in one second (FEV1% > 70%), stage 1 (FEV1% ≤ 70% and %FEV1 ≥ 80%), stage 2 (FEV1% ≤ 70% and %FEV1 50-79%), severe 3 (FEV1% ≤ 70% and %FEV1 30-49%), and stage 4 (FEV1% ≤ 70% and %FEV1 ≤30%).

Exercise tolerance was assessed using an exercise electrocardiogram (Master’s double two-step test). We categorized the patients into three groups: Group A, negative result in a standard method; Group B, negative result in a relaxed method (i.e. the patient could not tolerate the standard method, but could complete the exam by lowering the exercise burden); Group C, positive result or could not complete the exam. All scores and indices were calculated retrospectively in the current study as part of an exploratory and hypothesis-generating analysis. All statistical tests and plotting were performed in R (v.3.6.1; R Foundation for Statistical Computing, Vienna, Austria). The Kaplan-Meier method was used to calculate median overall survival.

## Results

Patients’ characteristics

Seven patients were included in the analysis. The baseline characteristics of the patients are summarised in Table 1.

**Table 1:** Patient background and characteristics ECOG PS, Eastern Cooperative Oncology Group Performance Score; CCI, Charlson Comorbidity Index; FEV1, Forced Expiratory Volume in one second; eGFR, estimated glomerular filtration rate; CRP, C -reactive protein; GPS, Glasgow Prognostic Score; GNRI, Geriatric Nutritional Risk Index; F, female; M, Male; HT, hypertension; IHD, ischemic heart disease; MGUS, Monoclonal gammopathy of undetermined significance; IP, interstitial pneumonia; DM, diabetes mellitus Reference ranges: eGFR (mL/min), 60.0-; Albumin (g/dL), 4.1-5.1; CRP (mg/dL), 0-0.14

Case #	Age (yrs)	Sex	Smoking history	ECOG PS	Past medical history	CCI risk group	FEV1％	%FEV1	%VC	eGFR (mL/min)	Albumin (g/dL)	CRP (mg/dL)	GPS	GNRI	Master's double two-step test
1	91	F	Never	1	HT, IHD, cerebral aneurism	Medium	67	152	145	45	3.6	0.1	0	No risk	Discontinued (knee pain)
2	90	M	Former	1	HT, MGUS	Medium	47	63	97	45.8	3.2	0.2	1	Moderate	Negative (Limited exercise)
3	93	M	Former	1	Stomach cancer & colon cancer (>5 years)	Medium	54	88	116	45.3	3.7	0	0	Moderate	Negative (Limited exercise)
4	90	M	Former	1	HT, IP (%DLCO 47%)	High	76	108	102	81.3	3.2	1.8	2	Moderate	Negative (Limited exercise)
5	96	Ｆ	Never	1	HT, dementia	High	74	121	101	32.3	4	0	0	No risk	Discontinued (T wave conversion)
6	94	M	Never	1	HT	Medium	53	77	101	57	3.6	0.06	0	Low	Negative
7	91	F	Never	1	HT, DM (HbA1c 6.8%), Rectal cancer (<5 years)	VeryHigh	68	107	103	45.7	3.7	0.67	0	No risk	Negative (Limited exercise)

Four patients were male, and three were female. The oldest patient was 96 years old. Three (43%) were former smokers. Six patients (86%) had a history of hypertension, and two (29%) had a prior history of cancer treatment. Five patients (71%) had obstructive pulmonary disorder. Six patients (86%) had stage three chronic kidney disease. Echocardiogram was performed in all patients, which showed sufficient cardiac functions. None of the patients harbored severe valve diseases. An exercise electrocardiogram was performed in all cases, although only one patient could complete the exam in a standardized manner. Four patients completed the exam by lowering the burden of exercise, and the remaining two patients failed to complete the exam due to knee pain and asymptomatic T wave conversion. 

Surgical details and postoperative courses

Surgical details and postoperative courses are summarised in Table 2.

**Table 2:** Surgical details and postoperative courses LN, lymph node; LUAD, lung adenocarcinoma; CT, computed tomography; OR, operating room

Case #	Pathological diagnosis	cStage (lung cancer, TNM v8)	pStage (lung cancer, TNM v8)	Side	Extent of resection	LN dissection	Localization method	Operation time (min)	Intraoperative bleeding (ml)	Adhesion
1	LUAD	IA1	IA2	Left	Wedge	-	-	80	0	>10cm^2^
2	LUAD	IA1	IA1	Left	Wedge	-	-	95	0	Majority of lung surface
3	LUAD (multiple)	IA2 & IA1	IA2 & IA1	Left	Wedge x2	-	Preoperative CT-guided hookwire	103	50	>10cm^2^
4	LUAD	IB	IVA (malignant pleural effusion)	Left	Lobectomy	Hilar LN	-	125	267	<10cm^2^
5	LUAD	IA2	IA2	Right	Wedge	-	-	45	30	<10cm^2^
6	LUAD	IA2	IA2	Right	Wedge	-	-	45	0	<10cm^2^
7	Rectal cancer metastasis	-	-	Left	Wedge x2	-	Intraoperative CT in hybrid OR	75	0	None

All patients underwent lung resection under 3-port VATS. Six patients (86%) were pathologically diagnosed as lung adenocarcinoma, and one patient had pulmonary metastasis of rectal cancer. Wedge resection was performed in six patients (86%), including two patients who underwent double wedge resections. A lobectomy was performed on one patient. Computed tomography (CT)-guided localization of the tumor was performed in two cases who underwent wedge resection. Intraoperative CT-guided localization was used for one patient using a hybrid operation room, while preoperative CT-guided hook wire localization was performed in another patient before the hybrid operating room became available. Mediastinal lymph node dissection or sampling was not performed for any case with lung cancer. Pleural adhesion was observed in six patients (86%), with one patient having extensive adhesion within the thoracic cavity.

Short-term and long-term outcomes after surgery

Postoperative outcomes are summarized in Table 3.

**Table 3:** Short-term and long-term outcomes after surgery POD, postoperative day

Case #	Postoperative complication	Chest tube removal (POD)	Hospital discharge (POD)	Adjuvant treatment	Last follow-up (days)	Status at last follow-up	Cause of death
1	None	2	5	None	1287	Alive	-
2	None	2	5	None	1496	Dead	Renal failure & heart failure
3	None	2	11	None	75	Alive	-
4	None	2	6	None	352	Dead	Lung cancer progression
5	Delirium	0	3	None	1215	Dead	Uncertain
6	None	1	5	None	1021	Alive	-
7	None	1	5	None	883	Alive	-

**Figure 1:**
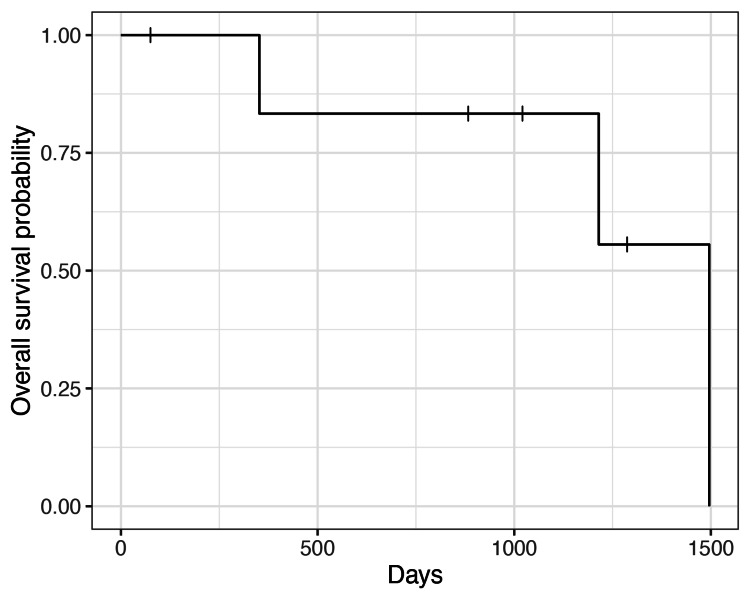
Kaplan-Meier curve for overall survival (N=7). The median overall survival after surgery was 1496 days (4.1 years).

The median duration of chest tube insertion was two days (range 0-2 days). One patient (case #5) who had a history of dementia developed postoperative delirium. None of the patients developed postoperative pneumonia. Six patients (86%) were discharged within one week after surgery, and the remaining one patient was discharged on postoperative day 11. To protect renal function, the usage of non-steroidal anti-inflammatory drugs was avoided. Nevertheless, postoperative pain could be well-tolerated, possibly due to the minimally invasive approach. All patients were discharged in stable condition without deterioration of their activities of daily living. Adjuvant chemotherapy was not given to any patients. Median overall survival was 1496 days (4.1 years) (Figure 1).

One patient (case #4), who had p-Stage IV lung adenocarcinoma, died 352 days after surgery due to lung cancer progression. Among the five patients who were followed up more than two years after surgery, none of the patients had a disease relapse. 

Conceptual framework of holistic evaluation for super-elderlies to assess and visualize the surgical risks

**Figure 2:**
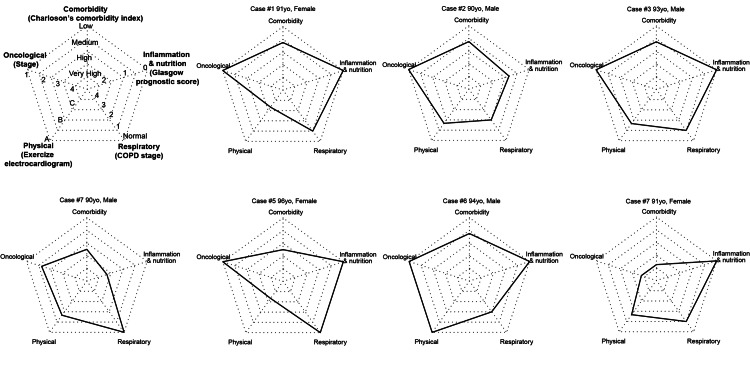
Example of radar plots visualizing multifaceted surgical tolerance and prognostic factors in each patient Multifaceted surgical tolerance and factors associated with prognosis are summarised in a radar plot with five axes: comorbidity axis (Charlson’s Comorbidity Index), inflammation and nutrition axis (Glasgow Prognostic Score), respiratory function axis (COPD stage), physical activity axis (exercise electrocardiogram), and oncological risk axis (clinical TNM stage).

A holistic assessment of each patient is crucial, especially in super elderly individuals. For the visualization of the holistic evaluation of each patient, the scores and groups used for risk evaluation were summarized in a radar plot with five axes: comorbidity, inflammation and nutrition, respiratory function, physical activity, and oncological stage (Figure 2).

For example, case #1 was characterized by impaired physical function. Due to chronic knee pain, she could not complete the exercise electrocardiogram. Therefore, the stress myocardial scintigraphy was performed to assess the cardiac function reserve further. The postoperative rehabilitation program was also customized by a physical therapist.

Case #4 had a normal respiratory function, and lobectomy was performed for her c-Stage IB non-small cell lung cancer (NSCLC). Unfortunately, the diagnosis of malignant pleural effusion could be confirmed only after the surgery, and it turned out to be Stage IVA. Retrospectively, impaired nutrition and inflammation depicted by GPS of 2 may have reflected the presence of advanced-stage cancer and poor prognosis. As depicted in the radar plots, all patients had at least one risk factor other than their age, even though most of these patients showed favorable short-term and long-term outcomes.

## Discussion

In this study, seven patients who were ≥90 years of age who underwent lung resection were retrospectively reviewed. Besides their super-high ages, all patients had multiple complications, including chronic kidney disease and obstructive respiratory disorder. Nevertheless, after lung resection under minimally invasive 3-port VATS, all patients could be discharged within two weeks without deterioration of their ADL. The median overall survival after surgery was 4.1 years. Of note, the patient with rectal cancer metastasis to the lung could also survive without disease burden for more than two years without additional treatment after pulmonary metastasectomy. These results suggest that minimally invasive surgery may yield favorable outcomes for the highly selected super-elderlies, and therefore, lung resection can be one of the treatment options for nonagenarians.

According to the life tables published by the Japanese government in 2023, the average life expectancy of a 90-year-old person is 4.2 years in males and 5.5 years in females [12]. Curative treatment for localized cancer may benefit a fraction of patients, even if they are in their 90s. It is crucial to minimize the post-treatment complications, especially in super-elderly individuals. If the patient can tolerate general anesthesia and one-lung ventilation, minimally invasive surgery may be one of the treatment options that can provide the chance of a cure.

Previous studies have shown that mediastinal lymph node dissection was significantly associated with postoperative complications in octogenarians [13,14]. Lack of mediastinal lymph node resection introduces the risk of missing pathological N2 diseases, which may be associated with poor long-term outcomes. Nevertheless, Nakao et al. recently reported that there was no survival benefit of mediastinal lymph node dissection in octogenarians with early-stage lung cancer using the Japanese nationwide prospective database and concluded that mediastinal lymph node dissection can be omitted in this patient group [15]. Likewise, Mimae et al. reported that overall survival after wedge resection and anatomical lung resection (segmentectomy and lobectomy) did not differ significantly in octogenarians with small-sized lung cancer (whole tumor sizes ≤2 cm and consolidation-to-tumor ratio >0.5) using the Japanese nationwide prospective database [16]. They also showed that overall survival after sublobar resection (wedge resection or segmentectomy) did not differ significantly in octogenarians with lung cancer >2 cm and ≤4 cm [17]. For super-elderly individuals, the highest priority would be to minimize the risk of surgical complications and maintain their ADL. Therefore, omitting mediastinal lymph node dissection and selecting wedge resection to reduce the operation time and maintain the residual lung volume may be justified even if a lobectomy with lymph node dissection is indicated for younger patients harboring similar disease. CT-guided localization of tumors, especially using intraoperative CT imaging, if available, may be utilized to accomplish successful wedge resection by localizing nonpalpable tumors, securing an adequate resection margin, and maximizing the residual lung volume [18]. 

Radiotherapy (RT) is another option for localized cancer. RT can improve survival for patients with unresected stage I or II NSCLC, and RT is currently considered the standard treatment for patients who are not medically fit or who prefer not to undergo surgery [19,20]. A pooled analysis of two randomized controlled studies comparing lobectomy and SBRT in patients with operable stage I NSCLC, which were both terminated due to poor patient recruitment, demonstrated no significant difference in the three-year recurrence-free survival rates between SBRT and surgery (86% vs. 80%) [21]. However, the optimal indication for surgery or RT remains unclear for operable super-elderly individuals, especially in the era of minimally invasive surgery.

Radar plot is a well-known method useful in visualizing multifaceted assessment [22,23]. Herein, we applied a radar plot to characterize each patient, with a special focus on surgical risks. Visualization of risk factors may help healthcare workers gain a holistic understanding of each patient and may benefit in preparing personalized care to minimize complications and enhance recovery after surgery. Although the prognostic scores used in the presented radar plots were arbitrarily chosen, the advantage of this method is that any prognostic scores or lab parameters can be chosen according to the risks and comorbidities that should be highlighted. For example, it might be helpful to add prognostic scores for postoperative pulmonary complications (e.g., the respiratory failure risk index [24] or ARISCAT risk scores [25]) to gain the focus of the healthcare workers in terms of the management of postoperative pneumonia. For the assessment of malnutrition, the Geriatric Nutritional Risk Index (GNRI) could also be used if serum albumin, height, and weight are available (Appendix) [11]. Alternatively, the Controlling Nutritional Status (CONUT) score may be applied if serum albumin and total cholesterol concentration and total peripheral lymphocyte count are available [26].

Limitations

This study has several limitations. This was a retrospective analysis of a very small number of patients who were highly selected as surgical candidates, which limited the statistical power and generalizability of the findings. The findings of the study are descriptive and cannot be generalized. In addition, life expectancy in Japan is longer than that of many other countries, and the nonagenarians in Japan may not be similar to those in other countries. Moreover, the absence of a control group makes it difficult to determine the true benefit of minimally invasive surgery in the study population. Whether the surgical resection under VATS truly benefits nonagenarians compared with open surgery or other treatment modalities, including RT, chemo-immunotherapy, and best supportive care, is unknown. Because all patients underwent 3-port VATS in our institution, comparisons between RATS and VATS, or between uniport and multiple ports, were out of the scope of the current study. The proposed radar plot of surgical risks in super-elderlies is an example of the conceptual framework to visualize the multifaceted assessment of each patient. The scoring methods used in the current manuscript were arbitrarily chosen. The tool is not verified for its utility and cannot be used for prognostication. It might be further refined by adding more aspects, such as cognitive functions and socioeconomic factors. Integration of validated multi-domain risk assessment tools such as the Preoperative Assessment of Cancer in the Elderly (PACE) may also enhance the holistic evaluation of each patient [27].

## Conclusions

Lung resection under a minimally invasive approach is feasible for carefully selected nonagenarians, yielding favorable short- and long-term outcomes. Lung resection via a minimally invasive approach may be a treatment option for selected cases of nonagenarians. Because super-elderly individuals likely harbor multiple comorbidities, a holistic assessment of each patient is important for personalized patient care.

A radar plot could summarize multifaceted surgical tolerance and factors associated with prognosis, which may be useful in visualizing each patient's holistic assessment. Although the scoring methods used in the radar plots were arbitrarily chosen, we envision that personalized patient care based on holistic evaluation may improve the treatment outcome by minimizing complications and enhancing recovery after surgery. However, because this study was a retrospective analysis of a small number of patients, the method should be validated and further refined. A larger-scale study involving multiple institutions would be necessary to increase statistical power and generalize the findings. Comparison with other treatment modalities would also be crucial to determine whether lung resection truly benefits the treatment outcomes of nonagenarians.

## Figures and Tables

**Figure 3:**
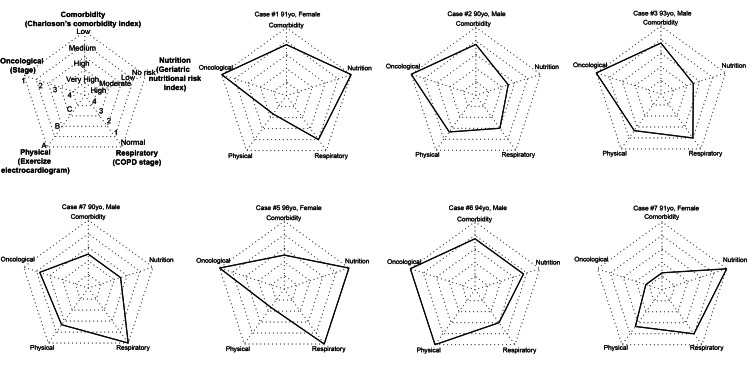
Alternative version of radar plots visualizing multifaceted surgical tolerance and prognostic factors in each patient. Multifaceted surgical tolerance and factors associated with prognosis are summarised in a radar plot with five axes: comorbidity axis (Charlson’s Comorbidity Index), nutrition axis (Geriatric Nutritional Risk Index), respiratory function (COPD stage), physical activity (exercise electrocardiogram), and oncological risk (clinical TNM stage).

## References

[1] (2025). Statistics Bureau of Japan. Result of the Population Estimates. https://www.stat.go.jp/english/index.html.

[2] (2025). Cancer Information Service, National Cancer Center, Japan. Cancer Statistics 2016-2020. https://ganjoho.jp/reg_stat/statistics/data/dl/excel/cancer_incidenceNCR(2016-2020)E.xlsx.

[3] (2025). Cancer Information Service, National Cancer Center, Japan. Cancer Statistics 1975-2015. https://ganjoho.jp/reg_stat/statistics/data/dl/excel/cancer_incidence(1975-2015)E.xls.

[4] Hori M, Matsuda T, Shibata A, Katanoda K, Sobue T, Nishimoto H (2015). Cancer incidence and incidence rates in Japan in 2009: a study of 32 population-based cancer registries for the Monitoring of Cancer Incidence in Japan (MCIJ) project. Jpn J Clin Oncol.

[5] Hino H, Murakawa T (2023). Lung cancer surgery for older patients: a narrative review. Curr Chall Thorac Surg.

[6] Yang CJ, Brown AB, Deng JZ (2020). The oldest old: A national analysis of outcomes for patients 90 years or older with lung cancer. Ann Thorac Surg.

[7] Miyazaki T, Yamasaki N, Tsuchiya T, Matsumoto K, Doi R, Machino R, Nagayasu T (2014). Pulmonary resection for lung cancer in nonagenarians: a report of five cases. Ann Thorac Cardiovasc Surg.

[8] Iwata T, Inoue K, Nishiyama N (2008). Lung cancer surgery in nonagenarians. Ann Thorac Cardiovasc Surg.

[9] Charlson ME, Pompei P, Ales KL, MacKenzie CR (1987). A new method of classifying prognostic comorbidity in longitudinal studies: development and validation. J Chronic Dis.

[10] Forrest LM, McMillan DC, McArdle CS, Angerson WJ, Dunlop DJ (2003). Evaluation of cumulative prognostic scores based on the systemic inflammatory response in patients with inoperable non-small-cell lung cancer. Br J Cancer.

[11] Bouillanne O, Morineau G, Dupont C (2005). Geriatric Nutritional Risk Index: a new index for evaluating at-risk elderly medical patients. Am J Clin Nutr.

[12] (2025). Abridged Life Tables for Japan 2023. https://www.mhlw.go.jp/english/database/db-hw/lifetb23/index.html.

[13] Okami J, Higashiyama M, Asamura H (2009). Pulmonary resection in patients aged 80 years or over with clinical stage I non-small cell lung cancer: prognostic factors for overall survival and risk factors for postoperative complications. J Thorac Oncol.

[14] Chida M, Minowa M, Karube Y, Eba S, Okada Y, Miyoshi S, Kondo T (2009). Worsened long-term outcomes and postoperative complications in octogenarians with lung cancer following mediastinal lymph-node dissection. Interact Cardiovasc Thorac Surg.

[15] Nakao M, Saji H, Mun M (2022). Prognostic impact of mediastinal lymph node dissection in octogenarians with lung cancer: JACS1303. Clin Lung Cancer.

[16] Mimae T, Saji H, Nakamura H (2021). Survival of octogenarians with early-stage non-small cell lung cancer is comparable between wedge resection and lobectomy/segmentectomy: JACS1303. Ann Surg Oncol.

[17] Mimae T, Saji H, Nakamura H (2023). Sublobar resection for non-small cell lung cancer in octogenarians: a prospective, multicenter study. Ann Thorac Surg.

[18] Fujiwara-Kuroda A, Aragaki M, Hida Y (2024). A simple and safe surgical technique for nonpalpable lung tumors: one-stop Solution for a nonpalpable lung tumor, Marking, Resection, and Confirmation of the surgical margin in a Hybrid operating room (OS-MRCH). Transl Lung Cancer Res.

[19] Wisnivesky JP, Halm E, Bonomi M, Powell C, Bagiella E (2010). Effectiveness of radiation therapy for elderly patients with unresected stage I and II non-small cell lung cancer. Am J Respir Crit Care Med.

[20] Shirai K, Aoki S, Endo M (2025). Recent developments in the field of radiotherapy for the management of lung cancer. Jpn J Radiol.

[21] Chang JY, Senan S, Paul MA (2015). Stereotactic ablative radiotherapy versus lobectomy for operable stage I non-small-cell lung cancer: a pooled analysis of two randomised trials. Lancet Oncol.

[22] Saary MJ (2008). Radar plots: a useful way for presenting multivariate health care data. J Clin Epidemiol.

[23] Stafoggia M, Lallo A, Fusco D, Barone AP, D'Ovidio M, Sorge C, Perucci CA (2011). Spie charts, target plots, and radar plots for displaying comparative outcomes of health care. J Clin Epidemiol.

[24] Arozullah AM, Daley J, Henderson WG, Khuri SF (2000). Multifactorial risk index for predicting postoperative respiratory failure in men after major noncardiac surgery. The National Veterans Administration Surgical Quality Improvement Program. Ann Surg.

[25] Canet J, Gallart L, Gomar C (2010). Prediction of postoperative pulmonary complications in a population-based surgical cohort. Anesthesiology.

[26] Ignacio de Ulíbarri J, González-Madroño A, de Villar NG (2005). CONUT: a tool for controlling nutritional status. First validation in a hospital population. Nutr Hosp.

[27] Pope D, Ramesh H, Gennari R (2006). Pre-operative assessment of cancer in the elderly (PACE): a comprehensive assessment of underlying characteristics of elderly cancer patients prior to elective surgery. Surg Oncol.

